# Supplementary figures and images for: Comparison of sonication with chemical biofilm dislodgement methods using chelating and reducing agents: Implications for the microbiological diagnosis of implant associated infection
Source: PLoS One. 2020 Apr 8;15(4):e0231389. doi: 10.1371/journal.pone.0231389 (PMC7141651; doi:10.1371/journal.pone.0231389)

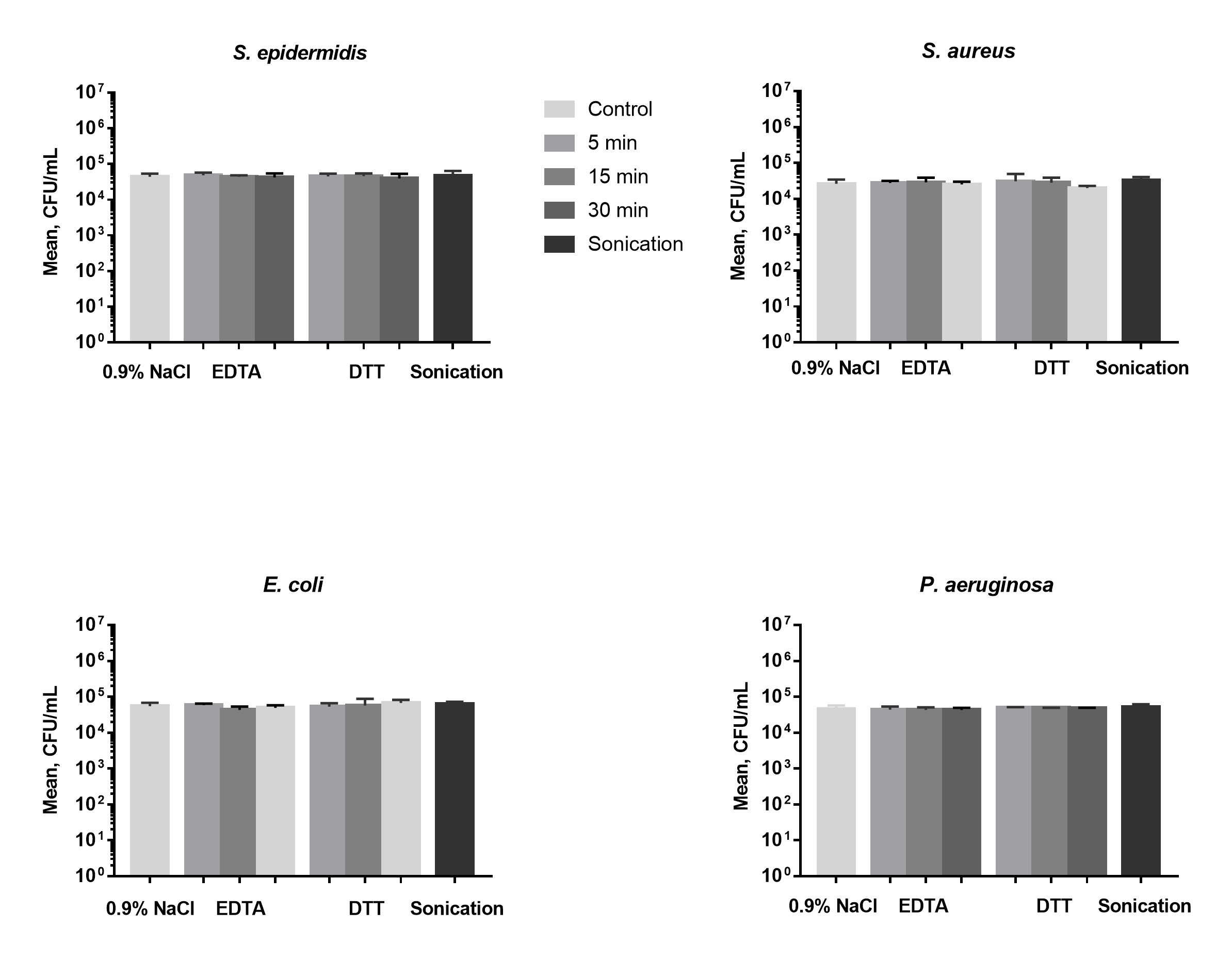

Supplement: S1 Fig — (TIF) [file pone.0231389.s001.tif]
