# Supplementary material for: Comparison of sonication with chemical biofilm dislodgement methods using chelating and reducing agents: Implications for the microbiological diagnosis of implant associated infection
Source: PLoS One. 2020 Apr 8;15(4):e0231389. doi: 10.1371/journal.pone.0231389 (PMC7141651; doi:10.1371/journal.pone.0231389)
Supplement: Attachment — Submitted filename: Response to Reviewers.docx [file pone.0231389.s002.docx]

**Karbysheva et al. Manuscript PONE-D-19-27719**

**Comparison of sonication with chemical biofilm dislodgement methods using chelating and reducing agents: potential implications for the microbiological diagnosis of implant associated infection**

Dear Editor

Thank you for the opportunity to re-submit our scientific work again to PLOS ONE for a potential review. Please find below our response to the comments of the Reviewers. Based on these comments, we have already revised our manuscript and hope that with this improved quality it may be suitable to send it for external review and potentially of interest for your prestigious Journal.

With best regards,

Andrej Trampuz

**Point-by-point rebuttal**

Scientific Editor Comments:

Reviewer #1:

1. The authors analyzed the activity (not efficacy as this is an in-vitro study) of chemical methods to be used for biofilm removal.

COMMENT: We corrected in the manuscript.

“We compared the activity efficacy of chemical methods for biofilm dislodgement to the standard sonication procedure in an established in vitro model of artificial biofilm.”

2. The manuscript is well written; the laboratory methods are suitable. The biofilm dispersion was determined by different methods. However, for this is a preliminary study. The purpose of removing biofilms from implants is medical microbiological diagnostics. For that, more microorganisms (incl. clinical strains) should be included.

COMMENT: Additionally we investigated two more microorganisms (*S. aureus* and *E. coli*).

3. EDTA may interfere with viability of microorganisms. The supplementary use of chemicals to sonication should included in that study.

COMMENT: We added supplementary material on the viability of planktonic bacteria in presence of chemical agents and sonication.

4. Minor remarks. Microorganisms associated with implant infections should be introduced.

COMMENT: We added in the manuscript additional information about microorganisms associated with implant infections.

“Most commonly isolated microorganisms in patients with periprosthetic joint infection are coagulase-negative staphylococci (30-45%) and Staphylococcus aureus (12-23%), followed by streptococci (9-10%), enterococci (3-7%), gram-negative bacilli (3-6%) and anaerobes (2-4%) [6]. Similar distribution of pathogens is observed in CIED [2] and neurosurgical shunt-associated infections [4].”

5. How do the glass beads for biofilm formation correspond to clinically used material?

COMMENT: We added the explanation and references in the method part.

“As a model to form the bacterial biofilm porous glass beads (diameter 4 mm, pore sizes 60 μm, ROBU®, Hattert, Germany) were used. Due to the high volume-to-surface ratio, glass beads were used for biofilm studies rather than smooth materials, as investigated in numerous previous research works regarding biofilm formation and anti-biofilm activity [15-20].”

Reviewer #2:

The authors performed an interesting in vitro study with the aim to evaluate the diagnostic performance of EDTA and DTT agents in comparison with the reference method (sonication) for bacterial dislodgement of S epidermidis and P aeruginosa biofilms.
Although the study has the following limitations:

1. only 2 strains were tested (lacking information on S aureus, which is a well known biofilm producer).

Reply: Additionally we investigated two more microorganisms (*S. aureus* and *E. coli*).

2. only reference strains were used,

COMMENT: Explained in the manuscript.

“We did not use clinical strains as they typically show larger variability and are not suitable for investigation of a new diagnostic method.”

3. only glass beads as foreign body materials,

COMMENT: We added the explanation and references in the method part.

“As a model to form the bacterial biofilm porous glass beads (diameter 4 mm, pore sizes 60 μm, ROBU®, Hattert, Germany) were used. Due to the high volume-to-surface ratio, glass beads were used for biofilm studies rather than smooth materials, as investigated in numerous previous research works regarding biofilm formation and anti-biofilm activity [15-20].”

It undoubtely confirmed the higher bacterial detection rate of sonication method compared with other techniques. This finding has important consequences in terms of the clinical application of these new techniques, which might be considered as complentary, and not alternative, to sonication.

4. Why 3 days of biofilm formation? Would the authors believe the results would be different with shorter or longer incubation time? Please comment on it

COMMENT: We incubated glass beads for 3 days for biofilm formation as described previously by Drago et al. Clin Orthop Relat Res. 2012. The authors grew the biofilm for 3 days until a visible biofilm was formed therefor further cultivation of mature biofilm is not needed.

5. The authors should also stress the potential use of sonication in implant infections other than orthopedics (ie intracardiac devices; neurosurgical shunts). Please add some references and a comment on it in the discussion.

COMMENT: We added the addition information in the manuscript.

“Implants Orthopedic devices are increasingly used to improve the mobility (joint replacement and bone fixation devices) in the treatment of degenerative joint disease (osteoarthritis) and for the fixation of bone fractures or enhance the survival and assist the performance of physiological functions (cardiac implantable electronic device (CIED) and neurosurgical shunts). Infections represent a significant complication of implant surgery, resulting in major challenges regarding the diagnosis and treatment [1-5].”

6. Insert a reference or an explanation on the use of porous glass beads as suitable model for biofilm formation in vitro in the method part.

COMMENT: We added the explanation and references in the method part.

7. Please add a reference or an explanation for the timing of EDTA and DTT exposition for biofilm dislodgement in the method part, not only in the discussion.

COMMENT: We added the explanation and references for the timing of EDTA and DTT exposition in the method part.

8. Furthermore, which is the rationale of choosing the EDTA and DTT concentrations? Please explain also in the method part.

COMMENT: We added the explanation of choosing EDTA and DTT concentration in the method part.

9. Insert the limit of detection in the method part (expressed as CFU/mL)

COMMENT: We corrected in the manuscript as CFU/mL.

10. Were the sonicated beads also submitted to vortexing process?

COMMENT: We did use the vortexing process in sonication protocol. We corrected the protocol in the method part.

“Briefly, each bead was inoculated in 1 ml 0.9% saline, vortexed for 30 sec, and sonicated at 40 kHz at intensity 0.1 Watt/cm2 (BactoSonic, BANDELIN electronic, Berlin, Germany) for 1 min and vortexed again for 30 sec.”

11. The viability experiment should be better explained or addressed in an appropriate reference.

COMMENT: We added supplementary material on the viability of planktonic bacteria in presence of chemical agents and sonication.

12. When comparing all the three quantitative different groups, was the ANOVA test performed? In the case the ANOVA test was not used, please indicate the reason.

COMMENT: We recalculated the result of the study using ANOVA test. New results are added in the manuscript.

13. Figure1 and Figure2; Figure5, 6. Please write per extenso EDTA and DTT. NS should be explained.

COMMENT: We corrected the information in the Figures.

14. Figure3 and figure4. Insert the different methods in the legend.

COMMENT: We corrected the information in the Figures.

15. Figure4. TTD should be explained. Which is the meaning of the arrow? Please explain.

COMMENT: We corrected the information in the Figures.

16. Figure3. 0,9% or 0.9%?. Please correct.

COMMENT: We corrected the information in the Figures.

17. Lines 151-153: the authors should describe that these CFUs obtained with these concentrations and timing derive from the above described experiments.

COMMENT: We corrected the information in the method part.

18. Line 155: insert CFU/mL

COMMENT: We corrected in the manuscript as CFU/mL.

18. Lines 156-157: at which concentration and timing of EDTA and DTT for P. aeruginosa? Please describe.

COMMENT: We corrected the information in the results part.

19. Lines 158-160: this sentence might be moved in a more appropriate place (ie, at the beginning of the paragraph).

COMMENT: We corrected the information in the results part.

20. Lines 173-177: the results of P aeruginosa are similar to those observed for S epidermidis. Please re-phrase the sentence and remove the term “although”, which might be confounding for the reader.

COMMENT: We corrected the information in the results part.

21. Lines 225-226. This sentence is not necessary since it has been already stated.

COMMENT: We corrected the information in the results part.

22. It would be interesting to have a comment from the authors regarding other non-culture based methods of diagnosis of implant associated infection (ie, the metabolic assay BTA, BioTimer Assay, which has been recently investigated in intracardiac and central venous catheters infections as a complementary method to sonication)
- Please include a sentence highlighting the limitations of the study

COMMENT: We added the additional information on non-culture based methods of diagnosis of implant associated infection in the limitation of the study.
