# Supplementary material for: Comparison of sonication with chemical biofilm dislodgement methods using chelating and reducing agents: Implications for the microbiological diagnosis of implant associated infection
Source: PLoS One. 2020 Apr 8;15(4):e0231389. doi: 10.1371/journal.pone.0231389 (PMC7141651; doi:10.1371/journal.pone.0231389)
Supplement: Attachment — Submitted filename: Response to Reviewers 2.docx [file pone.0231389.s003.docx]

**Karbysheva et al. Manuscript PONE-D-19-27719**

**Comparison of sonication with chemical biofilm dislodgement methods using chelating and reducing agents: implications for the microbiological diagnosis of implant associated infection**

Dear Editor

Thank you for the opportunity to re-submit our scientific work again to PLOS ONE for a potential review. Please find below our response to the comments of the Reviewers. Based on these comments, we have already revised our manuscript and hope that with this improved quality it may be suitable to send it for external review and potentially of interest for your prestigious Journal.

With best regards,

Andrej Trampuz

**Point-by-point rebuttal**

Scientific Editor Comments:

Reviewer #2

First revision:

2. only reference strains were used,

COMMENT: Explained in the manuscript.

“We did not use clinical strains as they typically show larger variability and are not suitable for

investigation of a new diagnostic method.”;

3. only glass beads as foreign body materials,

COMMENT: We added the explanation and references in the method part.

“As a model to form the bacterial biofilm porous glass beads (diameter 4 mm, pore sizes 60 μm,

ROBU®, Hattert, Germany) were used. Due to the high volume-to-surface ratio, glass beads

were used for biofilm studies rather than smooth materials, as investigated in numerous

previous research works regarding biofilm formation and anti-biofilm activity [15-20].”

Second revision, Reviewer #2: It undoubtedly confirmed the higher bacterial detection rate of sonication method compared with other techniques. This finding has important consequences in terms of the clinical application of these new techniques, which might be considered as complementary, and not alternative, to sonication.

These two limitations should be stated in the discussion. This in vitro analysis represents a fundament for further exploration in the clinical setting with clinical strains and real implants.

COMMENT: We stated these two limitations in the discussion.

“.., for biofilm formation we used only laboratory strains. Typically clinical strains show larger variability therefore to evaluate a new diagnostic method in vitro the laboratory strains are more suitable. Third, we used only porous glass beads for biofilm formation. The porous glass beads possess a high volume-to-surface ratio therefore this model to form bacterial biofilm is probably more suitable for in vitro biofilm investigation than smooth materials. The results derived from this in vitro analysis represent a fundament for further exploration in the clinical setting with clinical strains and real implants.”

First revision:

4. Why 3 days of biofilm formation? Would the authors believe the results would be different

with shorter or longer incubation time? Please comment on it

COMMENT: We incubated glass beads for 3 days for biofilm formation as described previously

by Drago et al. Clin Orthop Relat Res. 2012. The authors grew the biofilm for 3 days until a

visible biofilm was formed therefor further cultivation of mature biofilm is not needed.

Second revision, Reviewer #2: I agree with the reviewer. It remains unknown, whether the results would be different in more mature biofilms (as we are dealing with in the clinical setting). Comment this in the limitations setting.

COMMENT: We stated this limitation in the discussion.

“Forth, we incubated glass beads in the bacterial inoculum for 3 days until a visible biofilm was formed as described previously [14]. We assumed that further cultivation of mature biofilm to compare the ability of different methods for biofilm dislodgement is not needed. However it remains unknown, whether the ability of sonication or chemical methods for biofilm dislodgement would be different in more mature biofilms for example in the clinical setting when we deal with chronic implant-associated infections.”

First revision:

9. Insert the limit of detection in the method part (expressed as CFU/mL)

COMMENT: We corrected in the manuscript as CFU/mL.

Second revision, Reviewer #2: Could not find the detection limit in the methods part. Please make sure to provide the limit of detection.

COMMENT: We insert the limit of detection in the method part.

“The serial dilutions allowed to raise the upper limit of detection providing a reportable range from 0 to 100,000,000 CFU/mL.”

First revision:

14. Figure3 and figure4 . Insert the different methods in the legend.

COMMENT: We corrected the information in the Figures.

Second revision, Reviewer #2: Please mention the different methods tested in the legend.

COMMENT: Figure 3 is now Figure 2. We insert the methods in the legend.

First revision:

15. Figure4. TTD should be explained. Which is the meaning of the arrow? Please explain.

COMMENT: We corrected the information in the Figures.

Second revision, Reviewer #2: Please explain the meaning of the arrow in the legend

COMMENT: Figure 4 is now Figure 3. We explained the meaning of TTD in the legend.

Second revision, Reviewer #2: Please make sure, that the figures are numbered correctly and mentioned accordingly in the manuscript text. Figure 2 is now Fig 5, Fig 3 is now Fig 6, etc.

COMMENT: We revised the numbers of the figures and mentioned them correctly in the manuscript. We combined Figure 1 and Figure 2 in Figure 1. Figure 3 is now Figure 2. Figure 4 is now Figure 3. Figure 5 is now Figure 4. Figure 6 is now Figure 7. We added two additional Figures (5 and 6).

First revision: 18. Line 155: insert CFU/mL

COMMENT: We corrected in the manuscript as CFU/mL.

Second revision, Reviewer #2: Please add „CFU/ml“ in al the comparisons in the entire section (e.g. lines 170, 173, 178, 181)

COMMENT: We added “CFU/mL” in the manuscript.

Additional comments from Reviewer #2:

• Make sure to use the terms dislodgement (vs. dislodgment) and NaCl (vs. saline) uniformally throughout the manuscript

COMMENT: We corrected the terms in the manuscript.

• L 142: Please provide an introducing sentence before reporting the results. For example: „Figure 1 shows CFU counts of residual biofilms after exposure to chemical agents for variable durations.“

COMMENT: We added the sentence in the results part.
